# Imaging diagnosis of a rare case of intermittent intestinal pneumatosis: A consequence of ileocecal valve clip dysfunction?

**DOI:** 10.1016/j.radcr.2023.11.031

**Published:** 2023-12-05

**Authors:** Anna Russo, Vittorio Patanè, Carmine Zaccaria, Pasquale Verolino, Fabrizio Cioce, Francesco Stanzione, Alfonso Reginelli

**Affiliations:** a Department of Precision Medicine, University of Campania “L. Vanvitelli”, Naples, Italy; b Department of General and Emergency Surgery, Metabolic Care, Clinic Pineta Grande, Castel Volturno, Caserta, Italy

**Keywords:** Pneumatosis intestinalis, Postsurgery complications, Meckel diverticulum, Intermittent pneumatosis intestinalis, Pneumobilia, Laparoscopic cholecystectomy, Ileocecal valve dysfunction

## Abstract

Pneumatosis intestinalis is a condition characterized by the presence of gas or air pockets within the walls of the intestines. It can occur in any section of the gastrointestinal tract but it is most commonly found in the colon. Etiology and pathogenesis of PI are not yet fully understood, but several potential factors have been suggested to play a pivotal role in the development of this pathologic condition. Pneumatosis intestinalis seems to arise from a complex interplay between various factors, such as the integrity of the intestinal lining, pressure within the portal vein, the composition of the microbiological flora in the gut. Pneumatosis intestinalis can be caused by a variety of underlying conditions, such as bowel obstruction, intestinal ischemia, infection, inflammatory bowel disease, or certain medications. Symptoms may include abdominal pain, bloating, diarrhea, vomiting, and bloody stools. We present a case report of a 63-year-old male patient who underwent laparoscopic cholecystectomy for symptomatic cholelithiasis with recurrent cholecystitis. Following the surgery, the patient experienced a rapid drop in hemoglobin levels, necessitating an urgency regimen laparoscopic abdominal exploration which revealed Meckel's diverticulitis with active bleeding leading to diverticulectomy. The next day, the patient developed a radiological condition characterized by the co-presence of intermittent pneumatosis intestinalis, Portal pneumatosis and intermittent small bowel obstruction.

## Introduction

Pneumatosis intestinalis (PI) is a condition characterized by the presence of gas-filled collections within the wall of the digestive tract. They can be found throughout the digestive system, from the stomach to the rectum, although they are most frequently observed in the small intestine [1]. It can occur as a primary or secondary condition and may present with a range of symptoms [2]. Prompt diagnosis and appropriate management are essential for optimal outcomes.

Primary pneumatosis intestinalis, also known as idiopathic pneumatosis intestinalis, occurs without an identifiable cause. It is often seen in newborns and infants but can also occur in adults. The exact mechanism behind primary PI is unknown, but it is thought to be associated with bowel wall inflammation or ischemia [3].

Secondary pneumatosis intestinalis, on the other hand, is associated with a variety of conditions or factors. These include gastrointestinal disorders such as inflammatory bowel disease, intestinal obstruction, intestinal infections, and certain medications like immunosuppressants. Other underlying causes can include mechanical trauma to the bowel wall, such as due to surgery or endoscopy, as well as respiratory conditions like chronic obstructive pulmonary disease (COPD) that can cause gas to migrate into the digestive tract [4].

The clinical presentation of PI can vary depending on the severity and location of the gas collections [5,6]. Mild cases may be asymptomatic and only incidentally detected during imaging studies. However, in more severe cases, symptoms can include abdominal pain, bloating, distension, diarrhea, and rectal bleeding [7,8]. If the gas collection becomes extensive and causes bowel ischemia or perforation, a medical emergency can occur [9].

Diagnosis of PI is primarily done through imaging studies such as abdominal X-ray, computed tomography (CT), or ultrasound. These can reveal the presence of gas within the intestinal wall or lumen. In some cases, additional diagnostic procedures like endoscopy or biopsy may be required to determine the underlying cause [10].

Treatment of PI depends on the underlying cause and the severity of the condition [11]. Mild cases may not require any specific treatment, and the gas collections may resolve on their own. In more severe cases or when complications like bowel perforation occur, hospitalization and surgical intervention may be necessary. Management of the underlying condition, such as treating infections or adjusting medications, is also an important part of treatment [12].

Pneumatosis intestinalis can be associated with various conditions, including bowel ischemia, bowel obstruction, inflammatory bowel disease, infections, and certain connective tissue disorders [13]. The condition can be benign or life-threatening, depending on the underlying cause and severity. Of all cases of PI, 15% are classified as primary, whereas the remaining 85% are considered to be secondary [14]. Common clinical manifestations of pneumatosis intestinalis include abdominal pain (59%), diarrhea (53%), vomiting or nausea (14%), blood in the stools (12%), and mucus in the stools (12%) [15]. Secondary PCI is considered a symptom of an underlying disease. In the primary type, gas accumulation is a benign condition and results in the formation of an air-filled structure within the gut wall [16]. On the other hand, in secondary PI, is associated with a pathological underlying condition following ischemic and necrotic phenomena of bowel walls; gas distribution appears to be more linear [17]. PI is a relatively rare condition that is estimated to affect 0.03% of the global population. However, it is not a diagnosis in and of itself, but rather a radiological observation that can be associated with a wide range of medical conditions, ranging from relatively benign to potentially life-threatening conditions [18]. PI has been associated with collagen vascular disorders such as systemic sclerosis and inflammatory bowel disease, as well as the use of steroids and chemotherapeutic drugs. The cause of PI is not fully understood, but a common factor among cases linked to bronchial, mechanical, and bacterial causes is the disruption of mucosal integrity. Administration of corticosteroids over a prolonged period has been associated with mucosal atrophy and fibrosis in the intestines. The use of antimicrobial medications leads to a reduction in gas, and gas-filled cysts are often located close to blood arteries, providing support for the bacterial hypothesis that gas-producing bacteria, such as *Escherichia* and *Clostridia* species, may invade intraluminal compartments [19]. Blunt trauma can occur as a result of challenges experienced during surgeries or endoscopic procedures, leading to the release of gas from the digestive tract into the extraluminal area [20]. In lung diseases such as asthma, interstitial emphysema, and chronic bronchitis, alveolar rupture can cause the release of gas into the hepatic vasculature [21].

We present a case report of a 63-year-old male patient who went under laparoscopic cholecystectomy for symptomatic cholelithiasis with recurrent cholecystitis. Following the surgery, the patient experienced a rapid drop in hemoglobin levels, necessitating an abdominal CT scan with intravenous iodinated contrast.

## Case presentation

A 63-year-old male was admitted to the Radiology Department of University Hospital Luigi Vanvitelli in Naples for laparoscopic cholecystectomy due to a history of complicated biliary colic and cholecystitis. Shortly after the surgery, the patient experienced a rapid decline in hemoglobin levels, necessitating an emergency laparotomy to investigate potential iatrogenic source of active bleeding. During the exploratory laparotomy, a bleeding Meckel’s diverticulum was identified and surgically removed.

**Fig. 1:**
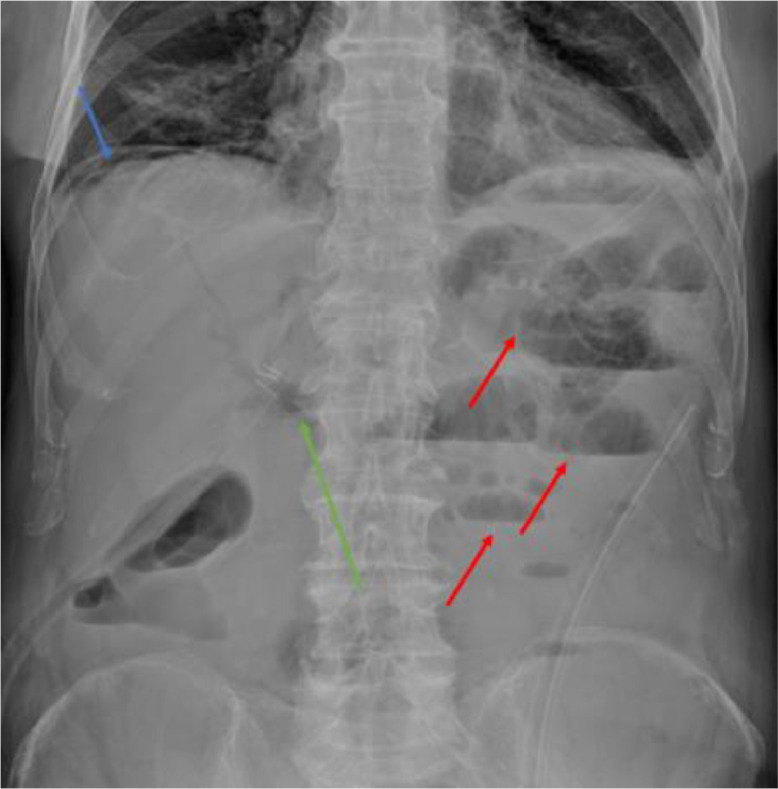
Abdominal X-ray performed 24 hours after surgical treatment for suspicious mechanical ileus showing subdiaphragmatic free air (blue arrow), a ladder-like pattern of fluid levels (red arrows), radiolucency corresponding to the biliary tree (green arrow).

**Fig. 2:**
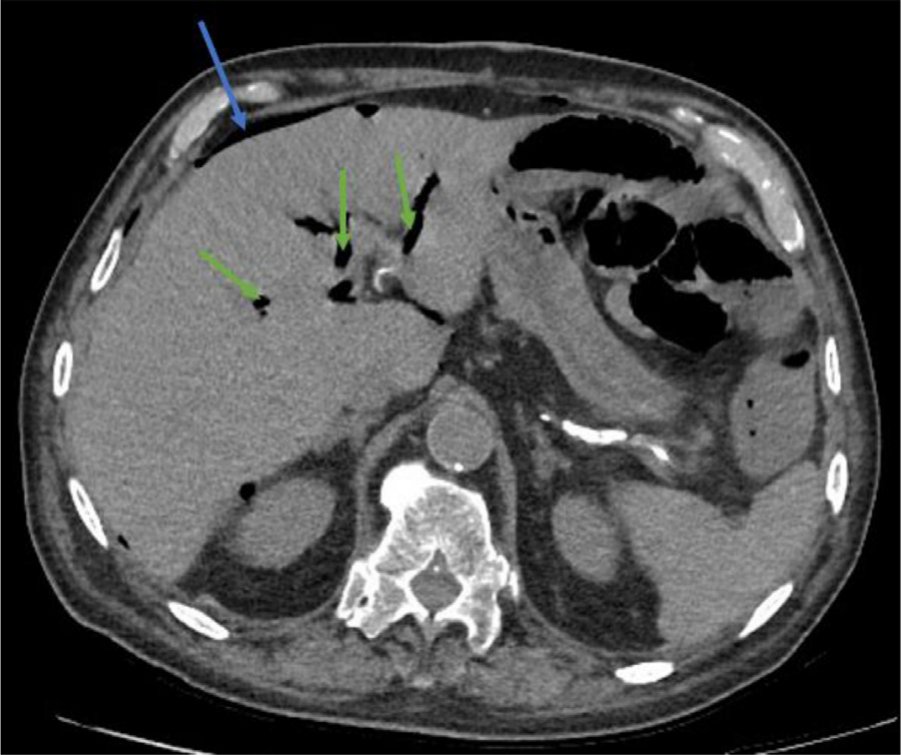
Noncontrast phase abdominal CT showing subdiaphragmatic free air (blue arrow), and air collection within the intrahepatic biliary tree.

**Fig. 3:**
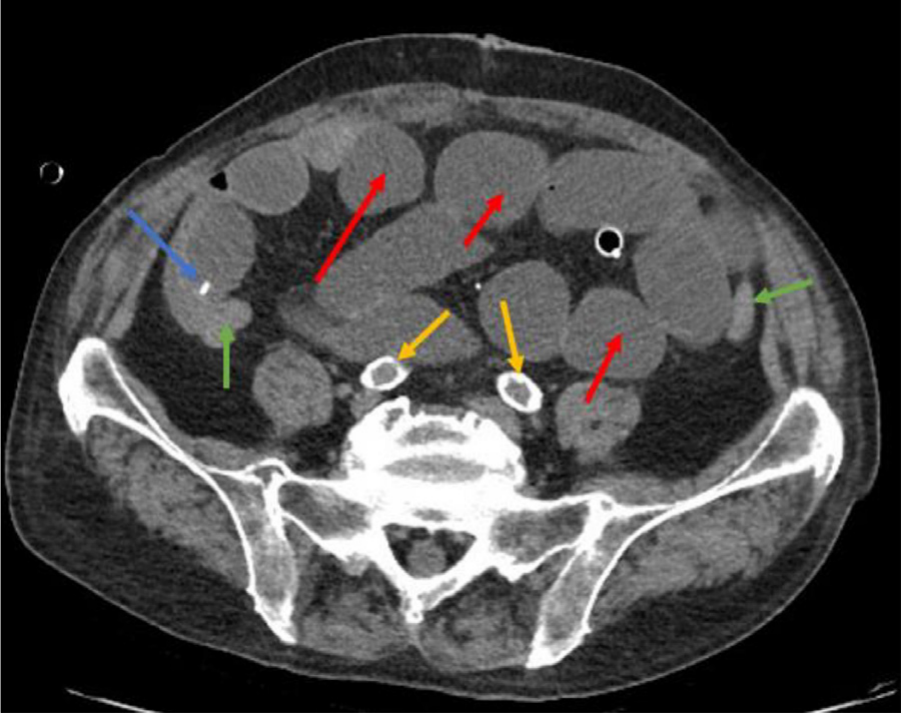
Noncontrast phase abdominal CT showing clips at the ileocecal valve (blue arrow), collapsed colonic walls (green arrows), and fluid-filled dilated ileal loops (red arrows) and diffuse calcified atherosclerosis involving the iliac arteries (yellow arrows).

Less than 24 hours after the surgery, the patient presented with diffuse abdominal pain, abdominal distension, and reduced intestinal peristalsis, raising suspicion of intestinal obstruction. An abdominal X-ray revealed subdiaphragmatic free air, a ladder-like pattern of fluid levels consistent with mechanical ileus, and radio transparency corresponding to the biliary tree (Fig. 1). A contrast-enhanced abdominal CT scan was performed to confirm these findings and explore potential causes. The CT scan revealed sub-diaphragmatic free air and pneumobilia (Fig. 2), clips at the ileocecal valve, collapsed colonic walls, and fluid-filled dilated ileal loops with abrupt caliber reduction at the site of the recent diverticulectomy clip (Fig. 3). The arterial phase of the CT scan demonstrated diffuse calcified atherosclerosis involving the aorta, superior mesenteric artery, and celiac trunk (Fig. 3).

**Fig. 4:**
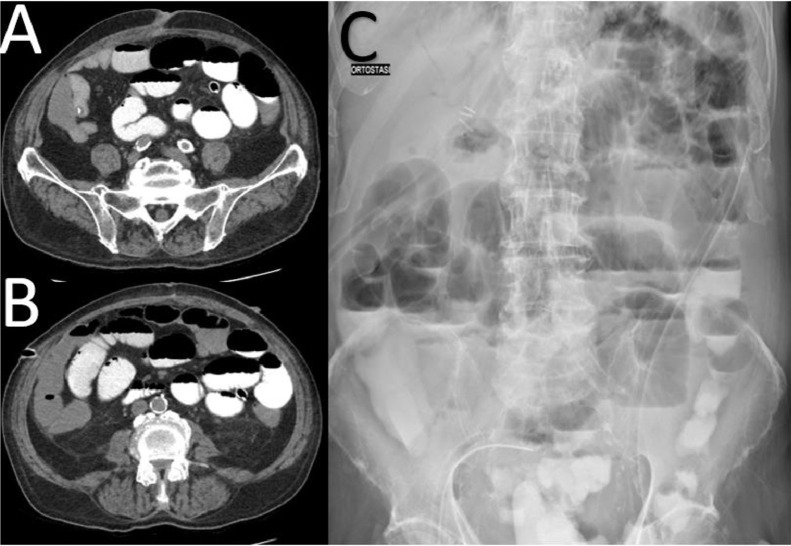
Oral contrast CT showing fluid and fluid-gas distensions in the ileum (A, B), with contrast passing beyond the ileocecal valve (C). Mild fluid distention of the right colon and sigmoid colon was also noted (C).

**Fig. 5:**
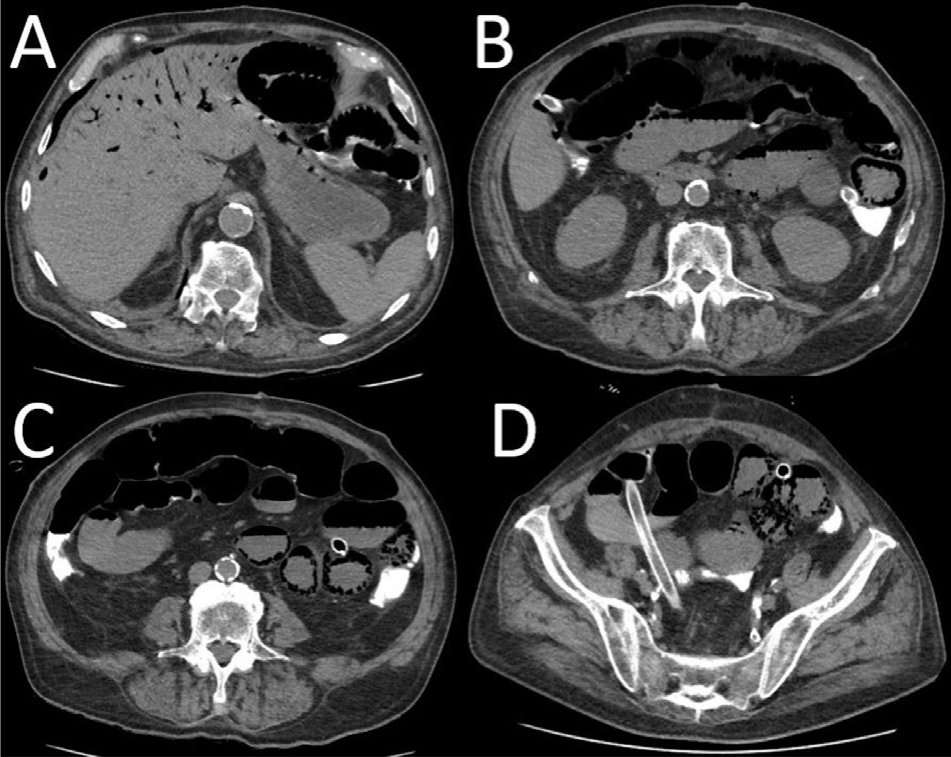
Oral contrast CT showing pneumatosis intestinalis (B-D) in different gut tracts and pneumobilia within intrahepatic biliary tree (A).

Given the stability of the patient and his comorbidities, a follow-up CT scan was scheduled 24 hours later, this time with oral contrast administration. The subsequent CT scan showed increased fluid and fluid-gas levels in the duodenum, jejunum, and ileum, with contrast passing beyond the ileocecal valve and presence of the surgical clips. Mild fluid distention of the right colon and sigmoid colon was also noted (Fig. 4). After another 24 hours of observation and supportive care, a repeat abdominal X-ray showed resolution of the obstructive pattern, with passage of the oral contrast beyond the ileocecal valve. However, areas of radiolucency within the hepatic parenchyma (intrahepatic pneumobilia) and between the intestinal walls were observed. A follow-up CT scan confirmed the resolution of the obstructive pattern while still demonstrating the presence of pneumobilia and intestinal pneumatosis (Fig. 5).

## Discussion

This case highlights a rare occurrence of intestinal pneumatosis and pneumobilia following laparoscopic cholecystectomy. The development of intestinal pneumatosis, characterized by the presence of gas within the intestinal walls, is a relatively uncommon finding, and its association with pneumobilia further adds to the complexity of the case. While the exact pathophysiology of these conditions in this particular case remains speculative, we hypothesize that dysfunction of the ileocecal valve clip played a role in the development of intestinal pneumatosis and subsequent pneumobilia. The presence of the surgical clips at the ileocecal valve raises the possibility of mechanical obstruction and impaired intestinal motility. This dysfunction could lead to stasis and distension of the intestinal lumen, creating a favorable environment for bacterial overgrowth and subsequent gas production. The resulting pneumatosis may have caused the escape of intramural gas nuclei through the compromised intestinal mucosa, leading to the observed pneumobilia.

Furthermore, the calcified atherosclerosis involving the aorta, superior mesenteric artery, and celiac trunk observed in the arterial phase of the CT scan suggests potential vascular compromise. Although it is unclear whether this atherosclerosis directly contributed to the development of intestinal pneumatosis, it may have impaired the intestinal blood supply, further exacerbating the compromised intestinal motility.

The resolution of the obstructive pattern and recurrent ileus seen in the follow-up imaging further support the hypothesis of ileocecal valve clip dysfunction as a contributing factor. The intermittent nature of the obstruction and its resolution suggest mechanical factors rather than a structural abnormality as the underlying cause. The subsequent resolution of symptoms and absence of recurrence after conservative management further support this hypothesis.

While this case report provides insight into a rare occurrence, further research and similar case reports are needed to establish a definitive causal relationship between ileocecal valve clip dysfunction and the development of intestinal pneumatosis and pneumobilia. Moreover, additional studies should explore potential predisposing factors, such as patient-specific anatomical variations or surgical technique, that may contribute to this complication. A better understanding of these factors will aid in the identification and management of similar cases in the future.

## Conclusions

This case report highlights the challenges encountered in managing a patient with intestinal pneumatosis complicated by portal pneumatosis. The successful use of conservative management in our patient, despite the poor prognosis indicated by radiological findings, emphasizes the importance of individualized treatment approaches based on the patient’s condition and contraindications. Further studies are warranted to elucidate optimal management strategies for similar cases.

Imaging diagnosis performed by integrating abdominal X-ray and contrast-enhanced CT with intravenous iodinated contrast allows for the diagnosis of intestinal complications, provides indications for follow-up, and identifies the presence of any post-surgical complications. The radiologist plays a crucial role in the procedural process and must therefore be aware of the type of surgery performed and the potential post-surgical complications. The clinical signs of abdominal discomfort and other nonspecific gut symptoms make it common for PI to be misdiagnosed as intestinal polyps, carcinoma, or colitis. As the number of surgeries increases, healthcare professionals must also increase their knowledge of PI. The diagnosis of PI heavily relies on the results of colonoscopy and abdominal CT. Treatment options for PI vary from observation and medication to oxygen therapy and surgery, depending on the patient’s symptoms and endoscopic findings. To avoid unnecessary surgery, the treatment approach should be tailored to the individual patient. If there are no significant complications, the prognosis is generally favorable.

## Patient consent

Informed written consent was obtained from the patient for publication of the case report and all imaging studies.
